# Supplementary material for: Activation of a nucleotide-dependent RCK domain requires binding of a cation cofactor to a conserved site
Source: eLife. 2019 Dec 23;8:e50661. doi: 10.7554/eLife.50661 (PMC6957272; doi:10.7554/eLife.50661)
Supplement: Supplementary file 1. [file elife-50661-supp1.docx]

| Protein | Organism | PDB Identifier |
| --- | --- | --- |
| MthK | *Methanothermobacter thermautotrophicus* | 1LNQ; 2AEF; 2AEJ; 2AEM; 2FY8;  2OGU; 3KXD; 3RBX; 3RBZ; 4EI2;  4L73; 4L74; 4L75; 4L76; 4RO0; |
| Kch | *Escherichia coli* | 1ID1 |
| KtrA | *Methanocaldococcus*  *jannaschii* | 1LSS |
| TrKA | *Vibrio parahaemolyticus* | 4J9U; 4J9V |
| TM1088A;  TM1088B | *Thermotoga maritima* | 3L4B; 2G1U |
| KtrA | *Bacillus subtilis* | 1LSU; 2HMS; 2HMT; 2HMU; 2HMV;  2HMW; 4J7C; 4J90; 4J91;5BUT |
| Kef | *Escherichia coli* | 3EYW; 3L9W; 3L9X |
| Kef | *Vibrio parahaemolyticus* | 3C85 |
| Kef | *Shewanella denitrificans* | 5NC8 |
| YbaL | *Escherichia coli* | 3FWZ |
| GsuK | *Geobacter sulfurreducens* | 4GVL; 4GX0; 4GX1; 4GX2; 4GX5 |
