# Supplementary material for: Activation of a nucleotide-dependent RCK domain requires binding of a cation cofactor to a conserved site
Source: eLife. 2019 Dec 23;8:e50661. doi: 10.7554/eLife.50661 (PMC6957272; doi:10.7554/eLife.50661)
Supplement: Supplementary file 2. — $ R16A-ADP, E125Q-ATP and E125Q-ADP have two different octameric rings in the crystal, indicated by (1) or (2). [file elife-50661-supp2.docx]

| **ATP** | **D36-D36 (Å)** | | | |
| --- | --- | --- | --- | --- |
| **Structure** | **Dimer 1** | **Dimer 2** | **Dimer 3** | **Dimer 4** |
| WT-ATP | 30.0 | 30.0 | 30.0 | 30.0 |
| R16K-ATP | 30.7 | 30.7 | 30.7 | 30.7 |
| R16A-ATP | 31.0 | 31.0 | 31.0 | 31.0 |
| WT-ATP-Ca | 30.7 | 30.7 | 30.7 | 30.7 |
| A80P-ATP | 32 | 31 | 31 | 32 |
| E125Q-ATP (1)^$^ | 33 | 34 | 33 | 34 |
| E125Q-ATP (2)^$^ | 31 | 32 | 31 | 32 |
| **ADP** | **D36-D36 (Å)** | | | |
| **Structure** | **Dimer 1** | **Dimer 2** | **Dimer 3** | **Dimer 4** |
| WT-ADP | 34.6 | 34.8 | 34.6 | 34.8 |
| R16K-ADP | 31.5 | 31.4 | 31.0 | 32.1 |
| R16A-ADP (1)^$^ | 31 | 32 | 30 | 32 |
| R16A-ADP (2)^$^ | 31 | 38 | 31 | 32 |
| A80P-ADP | 32 | 32 | 32 | 33 |
| E125Q-ADP (1)^$^ | 34 | 35 | 34 | 35 |
| E125Q-ADP (2)^$^ | 34 | 34 | 34 | 34 |
