# Supplementary material for: Activation of a nucleotide-dependent RCK domain requires binding of a cation cofactor to a conserved site
Source: eLife. 2019 Dec 23;8:e50661. doi: 10.7554/eLife.50661 (PMC6957272; doi:10.7554/eLife.50661)
Supplement: Supplementary file 3. — *Distances measured on the two faces of the ring are slightly different and we have named the front face as the one with the larger individual distance value. Values mentioned in the main text correspond to front face. # Non-square conformations were clustered in four groups according to L1/L2. $ R16A-ADP, E125Q-ATP and E125Q-ADP have two different octameric rings in the crystal, indicated by (1) or (2). [file elife-50661-supp3.docx]

|  | **L1/L2 (Å)** | |
| --- | --- | --- |
| **Octameric Ring** | Front face* | Back face* |
| **Square conformation** | | |
| WT-ATP | 30.0/30.0 | 29.0/29.0 |
| R16K-ATP | 30.8/30.8 | 30.3/30.3 |
| R16A-ATP | 29.9/29.9 | 29.0/29.0 |
| WT-ATP-Ca | 30.5/30.5 | 29.6/29.6 |
| **Non-square conformation 1^#^** | | |
| WT-ADP | 40.7/30.7 | 40.1/29.5 |
| **Non-square conformation 2^#^** | | |
| E125Q-ATP (1) ^$^ | 42/27 | 36/30 |
| E125Q-ADP (1) ^$^ | 42/27 | 36/30 |
| **Non-square conformation 3^#^** | | |
| R16A-ADP (1) ^$^ | 34/30 | 34/30 |
| R16A-ADP (2) ^$^ | 37/28 | 34/32 |
| E125Q-ATP (2) ^$^ | 34/30 | 34/30 |
| E125Q-ADP (2) ^$^ | 34/30 | 34/30 |
| **Non-square conformation 4^#^** | | |
| R16K-ADP | 52.0/21.9 | 51.3/22.5 |
| A80P-ATP | 52/23 | 51/22 |
| A80P-ADP | 52/22 | 52/21 |
