# Supplementary material for: Activation of a nucleotide-dependent RCK domain requires binding of a cation cofactor to a conserved site
Source: eLife. 2019 Dec 23;8:e50661. doi: 10.7554/eLife.50661 (PMC6957272; doi:10.7554/eLife.50661)
Supplement: Supplementary file 4. [file elife-50661-supp4.docx]

|  | ***B* factor (Å^2^)** | | | |
| --- | --- | --- | --- | --- |
|  | **R16K-ATP** | | **WT-ATP( 4J90)** | |
| **Atom** | Magnesium  refinement | Calcium  refinement | Magnesium  refinement | Calcium  refinement |
| Divalent cation | 74.32 | 109.75 | 125.14 | 165.00 |
| O from H_2_O (1) | 68.41 | 77.03 | - | - |
| O from H_2_O (2) | 66.67 | 76.92 | - | - |
| OE2 from E125 chain A | 84.88 | 92.19 | 90.83 | 89.69 |
| OE2 from E125 chain B | 92.66 | 102.90 | 103.50 | 102.36 |
| O2G from ATP 1 | 54.58 | 71.41 | 141.91 | 144.43 |
| O2G from ATP 2 | 90.22 | 105.68 | 157.40 | 151.27 |
| NH2 from R16 chain A | - | - | 93.14 | 100.24 |
| NH2 from R16 chain B | - | - | 93.62 | 91.41 |
