# Supplementary material for: Activation of a nucleotide-dependent RCK domain requires binding of a cation cofactor to a conserved site
Source: eLife. 2019 Dec 23;8:e50661. doi: 10.7554/eLife.50661 (PMC6957272; doi:10.7554/eLife.50661)
Supplement: Supplementary file 5. — Values shown are the mean ± SD values calculated from parameters from individual fits. Rate constants referred to in the main-text and figures were calculated as the mean of the inverse of τ extracted from individual fits. * For KtrAE125QB-ATP in 150 mM sorbitol the plateau had to be fixed during fitting since the signal is closer to a linear decay. [file elife-50661-supp5.docx]

| **Sorbitol external solution** | | | | | | | | |
| --- | --- | --- | --- | --- | --- | --- | --- | --- |
| **[Sorbitol]** | **Proteoliposomes** | **Amplitude** | **τ (s)** | **Amplitude (fast)** | **τ_fast_ (s)** | **Amplitude (slow)** | **τ_slow_ (s)** | **Plateau** |
| 150mM | KtrAB-ATP | 0.44±0.06 | 97.32±9.51 | - | - | - | - | 0.50±0.06 |
|  | KtrAB-ATP 2mM Mg | - | - | 0.39±0.03 | 10.40±0.88 | 0.12±0.02 | 110.34±25.17 | 0.25±0.06 |
|  | KtrAB-ADP | 0.31±0.01 | 291.42±33.11 | - | - | - | - | 0.64±0.01 |
|  | KtrAB-ADP 2mM Mg | 0.18±0.01 | 149.38±10.26 | - | - | - | - | 0.75±0.01 |
|  | KtrA_E125Q_B-ATP | 0.80±0.00 | 813.33±25.51 | - | - | - | - | 0.2* |
|  | KtrA_E125Q_B-ATP 2mM Mg | 0.39±0.01 | 270.00±4.85 | - | - | - | - | 0.59±0.01 |
|  | KtrA_R16A_B-ATP | 0.45±0.01 | 129.77±9.95 | - | - | - | - | 0.51±0.01 |
|  | KtrA_R16A_B-ATP 2mM Mg | - | - | 0.35±0.01 | 12.34±0.31 | 0.18±0.01 | 92.83±4.33 | 0.25±0.01 |
|  | KtrB-ATP | 0.44±0.07 | 288.96±41.69 | - | - | - | - | 0.54±0.07 |
|  | KtrB-ATP  2mM Mg | 0.28±0.01 | 108.33±2.21 | - | - | - | - | 0.66±0.01 |
|  | KtrB+KtrA-ATP | 0.24±0.03 | 159.60±3.05 | - | - | - | - | 0.75±0.02 |
|  | KtrB+KtrA-ATP 2mMg | - | - | 0.44±0.01 | 11.96±0.22 | 0.14±0.01 | 130.54±7.10 | 0.21±0.01 |
| **Choline Chloride external solution** | | | | | | | | |
| **[CholineCl]** | **Proteoliposomes** | **Amplitude** | **τ (s)** | **Amplitude (fast )** | **τ_fast_ (s)** | **Amplitude (slow)** | **τ_slow_ (s)** | **Plateau** |
| 150mM | KtrAB-ATP | - | - | 0.45±0.04 | 16.42±1.88 | 0.24±0.02 | 115.19±11.09 | 0.16±0.06 |
|  | KtrAB-ATP 0.7mM Mg | - | - | 0.43±0.04 | 17.17±0.64 | 0.24±0.02 | 126.36±5.40 | 0.17±0.04 |
|  | KtrAB-ATP 2mM Mg | - | - | 0.43±0.06 | 12.91±1.57 | 0.20±0.03 | 106.19±7.34 | 0.18±0.01 |
|  | KtrAB-ADP | 0.24±0.02 | 276.83±56.71 | - | - | - | - | 0.72±0.01 |
|  | KtrA_E125Q_B-ATP | 0.27±0.02 | 216.77±25.83 | - | - | - | - | 0.70±0.02 |
|  | KtrB-ATP | 0.25±0.04 | 193.01±19.11 | - | - | - | - | 0.72±0.05 |
|  | KtrA_R16A_B-ATP | - | - | 0.38±0.02 | 26.10±3.22 | 0.28±0.05 | 134.27±21.72 | 0.24±0.02 |
| 20mM | KtrAB-ATP | - | - | 0.38±0.06 | 30.55±5.55 | 0.25±0.07 | 153.07±25.75 | 0.32±0.02 |
|  | KtrAB-ATP 0.7mM Mg | - | - | 0.43±0.06 | 13.40±1.51 | 0.25±0.03 | 129.19±9.78 | 0.18±0.02 |
|  | KtrAB-ATP 2mM Mg | - | - | 0.39±0.02 | 10.57±0.80 | 0.17±0.04 | 116.49±11.33 | 0.20±0.01 |
|  | KtrAB-ATP 5mM Mg | - | - | 0.20±0.02 | 9.46±0.93 | 0.24±0.01 | 116.26±4.36 | 0.41±0.01 |
|  | KtrAB-ATP 0.7mM Ca | - | - | 0.33±0.01 | 11.81±1.03 | 0.24±0.01 | 120.28±7.69 | 0.24±0.01 |
|  | KtrAB-ATP 2mM Ca | - | - | 0.38±0.02 | 10.93±0.14 | 0.21±0.01 | 130.42±5.24 | 0.21±0.01 |
|  | KtrAB-ATP 5mM Ca | 0.19±0.01 | 97.99±3.69 | - | - | - | - | 0.66±0.01 |

| **Choline Acetate external solution** | | | | | | | | |
| --- | --- | --- | --- | --- | --- | --- | --- | --- |
| **[Choline Acetate]** | **Proteoliposomes** | **Amplitude** | **τ (s)** | **Amplitude (fast)** | **τ_fast_ (s)** | **Amplitude (slow)** | **τ_slow_ (s)** | **Plateau** |
| 150mM | KtrAB-ATP | - | - | 0.33±0.01 | 23.54±1.09 | 0.31±0.01 | 143.83±8.73 | 0.26±0.01 |
| **Lithium Chloride external solution** | | | | | | | | |
| **[LiCl]** | **[LiCl]** | **Amplitude** | **τ (s)** | **Amplitude (fast)** | **τ_fast_ (s)** | **Amplitude (slow)** | **τ_slow_ (s)** | **Plateau** |
| 150mM | KtrAB-ATP | 0.39±0.03 | 229.34±10.39 | - | - | - | - | 0.50±0.03 |
| 5mM | KtrAB-ATP | - | - | 0.31±0.04 | 47.56±5.33 | 0.31±0.03 | 222.95±30.25 | 0.34±0.01 |
|  | KtrAB-ATP 0.7mM Mg | - | - | 0.35±0.04 | 14.47±0.24 | 0.31±0.03 | 149.63±5.32 | 0.21±0.01 |
| 20mM | KtrAB-ATP | - | - | 0.31±0.03 | 21.85±0.63 | 0.36±0.04 | 178.09±5.70 | 0.27±0.01 |
|  | KtrAB-ATP 0.7mM Mg | - | - | 0.25±0.02 | 14.28±0.37 | 0.37±0.01 | 164.03±3.75 | 0.28±0.01 |
|  | KtrB-ATP | 0.38±0.03 | 298.46±23.10 | - | - | - | - | 0.60±0.04 |
|  | KtrA_E125Q_B-ATP | 0.39±0.01 | 272.15±13.48 | - | - | - | - | 0.58±0.01 |
